# Contributing Factors of Dielectric Properties for Polymer Matrix Composites

**DOI:** 10.3390/polym15030590

**Published:** 2023-01-24

**Authors:** Quan Wang, Junbo Che, Weifei Wu, Zhendong Hu, Xueqing Liu, Tianli Ren, Yuwei Chen, Jianming Zhang

**Affiliations:** 1 Key Laboratory of Rubber-Plastics, Ministry of Education/Shandong Provincial Key Laboratory of Rubber-Plastics, Qingdao University of Science & Technology, Qingdao 266042, China; 2 Key Laboratory of Optoelectronic Chemical Materials and Devices, Ministry of Education and Flexible Display Materials and Technology Co-Innovation Centre of Hubei Province, Jianghan University, Wuhan 430056, China; 3 Mississippi Polymer Institute, The University of Southern Mississippi, Hattiesburg, MS 39406, USA

**Keywords:** polymer composites, dielectric properties, microstructure, functional polymers

## Abstract

Due to the trend of multi-function, integration, and miniaturization of electronics, traditional dielectric materials are difficult to satisfy new requirements, such as balanced dielectric properties and good designability. Therefore, high dielectric polymer composites have attracted wide attention due to their outstanding processibility, good designability, and dielectric properties. A number of polymer composites are employed in capacitors and sensors. All these applications are directly affected by the composite’s dielectric properties, which are highly depended on the compositions and internal structure design, including the polymer matrix, fillers, structural design, etc. In this review, the influences of matrix, fillers, and filler arrangement on dielectric properties are systematically and comprehensively summarized and the regulation strategies of dielectric loss are introduced as well. Finally, the challenges and prospects of high dielectric polymer composites are proposed.

## 1. Introduction

As the demand for electronics and capacitor devices increases, high dielectric materials have attracted increasing attention [1,2,3]. Polymer materials own advantages of ease of processing, flexibility, and good mechanical properties but the dielectric properties are usually less than satisfactory. Therefore, the preparation of high dielectric composites by introducing high dielectric fillers has become a research hotspot [4,5]. Fillers or interfaces can be easily polarized under external electric fields then enhances the dielectric permittivity of polymer composites [6]. Compared with traditional dielectric materials, high dielectric polymer composites offer more benefits, such as easy processing, excellent mechanical properties, and good flexibility [7].

The dielectric permittivity (*ε*) and dielectric loss (tanδ) composes the dielectric properties of the composites [8]. Additionally, the *ε* is composed by a real (*ε*′) and imaginary part (*ε*″). The dielectric loss refers to the phenomenon of heat generation accompanied by energy consumption during polarization [8,9,10]. The relationship between the dielectric permittivity (ε) and dielectric loss (tanδ) is shown by the following Equation (1):

(1) $$tan\delta =\frac{\varepsilon \prime \prime }{\varepsilon \prime }$$

where *ε*′ is the real permittivity of the system and *ε*″ is the imaginary permittivity of the system. Dielectric loss is mainly attributed to polarization loss and conductivity loss. Polarization loss is mainly generated by the polarization of the molecular dipole. Polarization loss occurs during the polarization and relaxation process, which inevitably consumes electrical energy to overcome internal viscous resistance of the medium, thus resulting in dielectric loss of the material. Any movement of current carriers, even in very restricted areas, also consumes energy to overcome the resistance and results in conductivity loss [6]. Both the dielectric permittivity and dielectric loss directly affect the practical application of dielectric materials [11,12]. By the reasonable selection principle of the matrix and filler, dielectric properties of composites are manipulated. For instance, the addition of conductive fillers or ceramic fillers can increase the dielectric permittivity. By introducing insulating fillers or core-shell structures to block the formation of conductive paths, the increase of dielectric loss can be effectively suppressed [13,14,15]. Of course, not all fillers can enhance dielectric permittivity, the dielectric permittivity decreases when fillers such as POSS with a cage structure are introduced [16,17,18]. 

**Figure 1:**
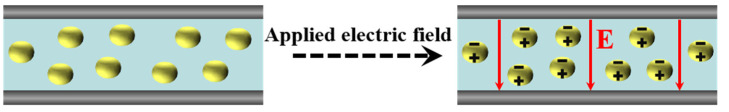
Polarization under electric fields.

Based on physics theory, polarization can affect the dielectric permittivity of the material and the charge of the material accumulates under the external electric filed leading to the polarization phenomenon (Figure 1). Factors affecting dielectric permittivity include electron polarization, atomic polarization, dipole polarization, and interfacial polarization [6,19,20]. Both electronic polarization and atomic polarization are collectively referred to as deformation polarization or induced polarization. Polymers are subject to deformation polarization or induced polarization in the high-frequency region. Interfacial polarization is generated by the aggregation of electrons or ions in the dielectric at the non-homogeneous interface and due to the different polarization rates of the components on either side of the interface, this often occurs at the interfaces of impurities, defects, crystalline, and amorphous regions [21,22,23,24]. Compared to the three polarization phenomena mentioned above, dipole polarization usually takes a longer time since the molecules are required to override inertia and resistance during polarization; therefore, dipole polarization occurs at a low-frequency range. The above mentioned four types of polarization determine the dielectric permittivity of the material. The relationship between polarization and dielectric permittivity is as follows:

(2) $$P=\left(\varepsilon_{r}-1\right)\varepsilon_{0}E$$

where *P* is the polarization intensity and *ε_r_* and *ε*_0_ represents the dielectric permittivity of the material and vacuum, respectively. *E* is the strength of applied electric fields. It can be derived that the polarization intensity and dielectric permittivity are proportional to each other based on Equation (2). Hence, the higher the polarity of the material the higher its dielectric permittivity. This is also the cause of why the polymer owning of a large number of polar groups are chosen as the matrix of high dielectric polymer composites. Table 1 shows dielectric properties of common matrix at 1 KHz frequency [6].

Compared with the principle of matrix selection, the selection strategy of fillers for high dielectric polymer composites have to take more factors into consideration, including polarity, electrical conductivity and interfacial effects, processing properties, and mechanical properties [43]. The commonly used fillers are classified as the following: (1) conductive fillers [44,45,46,47]; (2) ceramic fillers; and (3) polar polymer fillers [48,49,50,51,52,53,54,55]. The addition of fillers can increase the dielectric constant of composites to some extent; however, it also increases the dielectric loss simultaneously, which is undesirable for practical applications.

**Figure 2:**
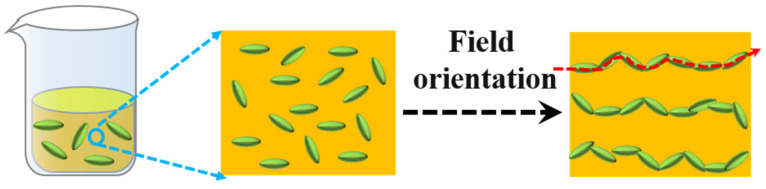
Fillers oriented under external electric fields.

In addition to the selection strategies for fillers, controlling the distribution of fillers by external fields can also improve the dielectric permittivity of the composite. External fields can align the particles by shearing force or electrophoretic force along one direction in the polymer matrix (Figure 2). This alignment structure effects the dielectric properties on many aspects. Based on the above considerations, this review systematically discusses the impact on fillers, structural design on dielectric permittivity of composites, as well as regulation strategies of dielectric loss. Typically used fillers of a different nature, such as conductive, inorganic, and organic, have been investigated. At the end, the challenges and prospects of high dielectric polymer composites are proposed [55,56,57,58]. The dielectric properties of polymer high dielectric composites are mainly affected by the matrix and filler. Distinct from reported reviews, in this review, we introduce not only the effects of polymer matrix and filler type but also the cutting-edge research, such as the effect of the distribution method of filler on the dielectric properties of composites and the up-to-date method of suppressing the dielectric loss of the composites. We Hope that this review article will give readers a comprehensive understanding and inspire future multidisciplinary research efforts in high dielectric polymer composites arena.

## 2. Influence of Polymer Matrix on Dielectric Properties of Composites

Polymer materials are widely used in electronic fields, such as integrated circuit boards, film capacitors, and display screens, due to their excellent processability and low dielectric loss. Due to the huge demand, a variety of polymer materials have gradually emerged, including polystyrene, polyethylene, polycarbonate, polyvinylidene fluoride, and other materials [59,60,61,62]. 

**Figure 3:**
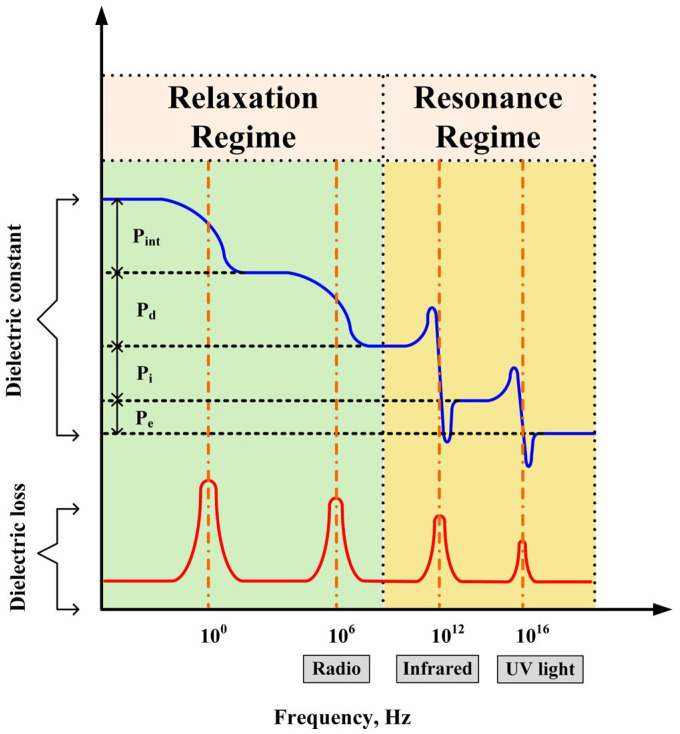
Polarization types at different frequencies. P_e_, P_i_, P_d_, and P_int_ refer to e-polarization, ion-polarization, dipole-polarization, and interfacial polarization, respectively. Adapted with permission [6]. Copyright 2016, American Chemical Society.

As shown in Table 1, polymers with high polarity are preferred for high dielectric polymer composites matrix [6]. The most commonly used include PVDF, PMMA, etc., while polyvinyl alcohol has a high dielectric permittivity, but its loss is also very high, affecting the practical application, so it is generally not used as a matrix. PTFE, BOPP, LDPE, HDPE, and PS have high molecular regularity and there are no polar groups in the molecular chain. Therefore, these materials own low dielectric permittivity and dielectric loss. The polar groups in the system will improve the dielectric permittivity and dielectric loss of the composite, such as PVDF, PVA, etc. Of course, the same material’s dielectric permittivity and dielectric loss are not invariable; the different test frequency will also cause the change of dielectric permittivity. Figure 3 shows that different polarization phenomena will occur at different test frequencies and accumulate continuously [6]. Therefore, dielectric permittivity and dielectric loss for a certain test frequency may be a result of multiple polarization phenomena superimposed.

**Figure 4:**
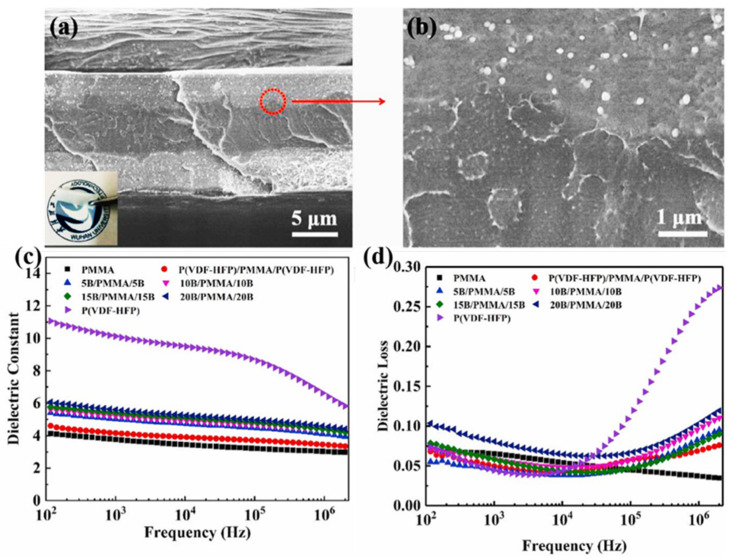
(**a**) SEM diagrams for sandwich structure composite with 10 wt% BT-NPs. (**b**) SEM images for adjacent region of PVDF-HFP/BT-NPs and PMMA. The dielectric permittivity (**c**) and dielectric loss (**d**). Adapted with permission [65]. Copyright 2021, Elsevier.

The polymer matrix can provide excellent mechanical properties for the composite and also has a great influence on the dielectric properties of the composite. The dielectric properties of composite materials are greatly influenced by the matrix [63,64]. As shown in Figure 4, the dielectric composite with the middle layer of polymethyl methacrylate (PMMA) and the outer layer of polyvinylidene fluoride-co-hexafluoropropvlene (PVDF-HFP) filled with BaTiO_3_ nanoparticles (BT-NPs) was prepared (Figure 4a,b) [65]. The dielectric properties of PMMA, PVDF-HFP, PVDF-HFP/PMMA/PVDF-HFP, and the designed sandwich composites (PVDF-HFP/BT-NPs)/PMMA/ (PVDF-HFP/BT-NPs) were characterized. The dielectric permittivity of P(VDF-HFP) was around 11 (at 100 Hz), while that of PMMA was only 4. The dielectric permittivity of the sandwich structure composite is 4.8 (Figure 4c). The dielectric permittivity of the sandwich structure composite could be enhanced to around 6 by adding 20 wt% BaTiO_3_ nanoparticles, but the dielectric permittivity of PVDF-HFP was still higher than that of the composite. In addition, the addition of BaTiO_3_ nanoparticles into PMMA also caused the increase of dielectric loss (Figure 4d), which is undesirable. Therefore, the effect of polarity on the dielectric permittivity of the polymer is pronounced. There are a large number of polar groups in the PVDF matrix, and PVDF has a higher dielectric constant than PMMA and the difference is very large. In other words, a polymer matrix with weak polarity is not always able to exceed a polymer matrix with strong polarity by adding a certain content of filler. Therefore, the selection of matrix is very important for high dielectric composites [63,64]. Commonly, polymers with strong polarity are preferred when designing high dielectric composites.

## 3. Influence of Fillers on Dielectric Properties of Polymer Composites

Filler has a great influence on the performance of composites as well. Usually, fillers with high polarity or conductivity can be added to greatly increase the dielectric permittivity of the composite. However, the dispersion is crucial to both the dielectric permittivity and dielectric loss [66,67,68,69]. Poor dispersion leads to higher dielectric loss and lower dielectric permittivity. Moreover, the addition of filler will inevitably generate a mass of interface in the composite, which leads a troublesome issue of increasing dielectric loss [70,71,72]. It is worth mentioning that the interaction between the filler and the matrix may restrict the movement of the molecular chains, which can reduce dielectric loss [73]. In high dielectric composite systems, conductive fillers, ceramic fillers, and polar polymer fillers are the mainly used categories. Their advantages and disadvantages were summarized in the Table 2.

### 3.1. Application of Conductive Fillers in Dielectric Composites

Conductive fillers are mainly divided into metallic materials and carbon materials. Metallic particles include silver, copper, aluminum, and nickel and carbon materials involve carbon nanotubes, graphene, carbon black, etc. [74,75,76,77,78]. Although all these particles above mentioned can greatly increase the dielectric permittivity of composites, their disadvantages cannot be ignored. On the one hand, the conductive fillers are less compatible with polymer matrix, accompanying agglomeration phenomenon, which has bad influence on both processing and performance of the composites [10]. On the other hand, the fillers contact each other and form conductive pathways easily due to their good conductivity, which results in current leakage and increases the dielectric loss of the composites [43]. Therefore, many researchers have addressed the drawbacks of poor filler-matrix compatibility by modifying conductive fillers. For instance, the compatibility problem can be solved by modifying the conductive filler with a insulative polymer layer, and if the layer is isolating, the formation of the conductive pathway can also be blocked [79,80].

**Figure 5:**
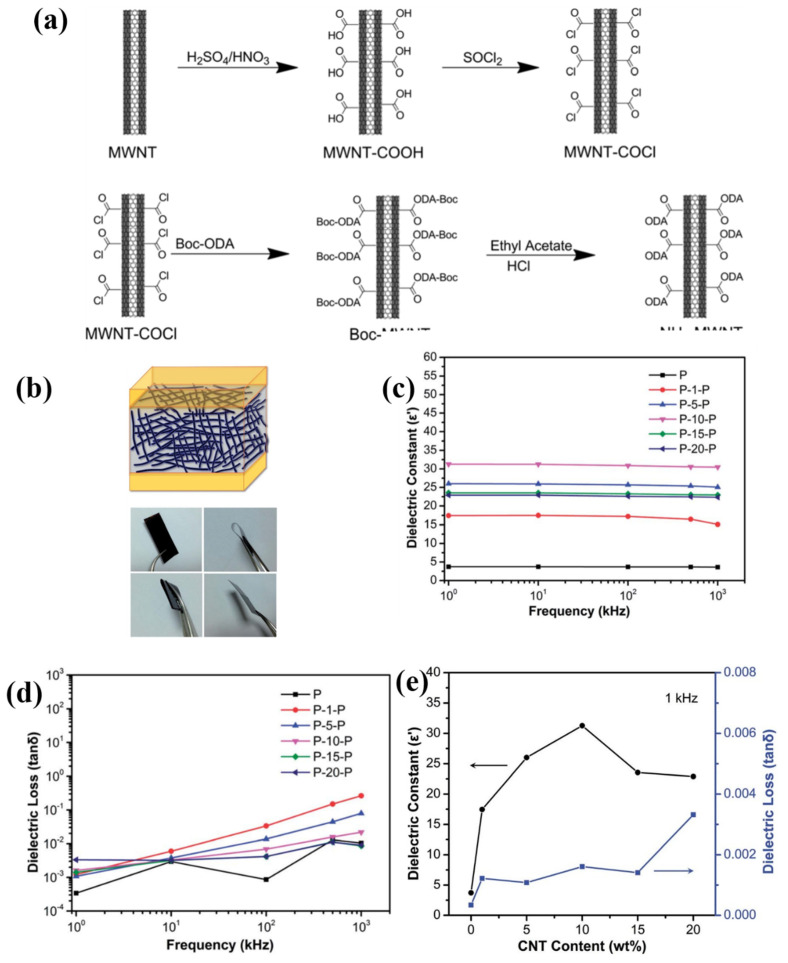
(**a**) Synthetic route of NH_2_-MWNT. (**b**) Schematic and physical diagram of NH_2_-MWNT/PI composites. (**c**) Dielectric permittivity and (**d**) dielectric loss of NH_2_-MWNT/PI composite films. (**e**) The dielectric properties of the composites at 1 KHz influenced by the NH_2_-MWNT content. Adapted with permission [81]. Copyright 2014, Royal Society of Chemistry.

Chen et al. prepared carboxylated multiwalled carbon nanotubes using acid oxidation and then acyl chlorinated carbon nanotubes (NH_2_-MWNT) were prepared by immersing the carboxylated multiwalled carbon nanotubes into chlorinated sulfoxide [81]. Then, NH_2_-MWNT/PI composites were prepared by using modified carbon nanotubes as fillers and polyimide (PI) as the matrix (Figure 5a,b). The dielectric permittivity of composites gradually increases with increasing NH_2_-MWNT content, which could reach up to 31 (1 KHz) until the content of NH_2_-MWNT was 10 wt%, and the dielectric loss is only 0.022. The dielectric permittivity of the composites began to decrease when the content of NH_2_-MWNT was over 10 wt%. That is because NH_2_-MWNT has good dispersion in the composite by surface modification when the content of NH_2_-MWNT was not exceeding 10 wt%, while the dispersion of NH_2_-MWNT became worse as long as the content was over the limit, which leads to a decrease in dielectric permittivity and an increase in dielectric loss (Figure 5c–e). Carbon nanotubes have excellent electrical conductivity, which can increase the polarization phenomenon and the dielectric constant of composites. The reduction of mutual contact of carbon nanotubes after modification can suppress the elevated dielectric loss to some extent. Therefore, the method of modifying conductive fillers by polymer materials can improve the compatibility between the conductive fillers and polymer matrix to a certain extent and also can restrain the formation of conductive pathways.

### 3.2. Application of Ceramic Fillers in Dielectric Composites

**Figure 6:**
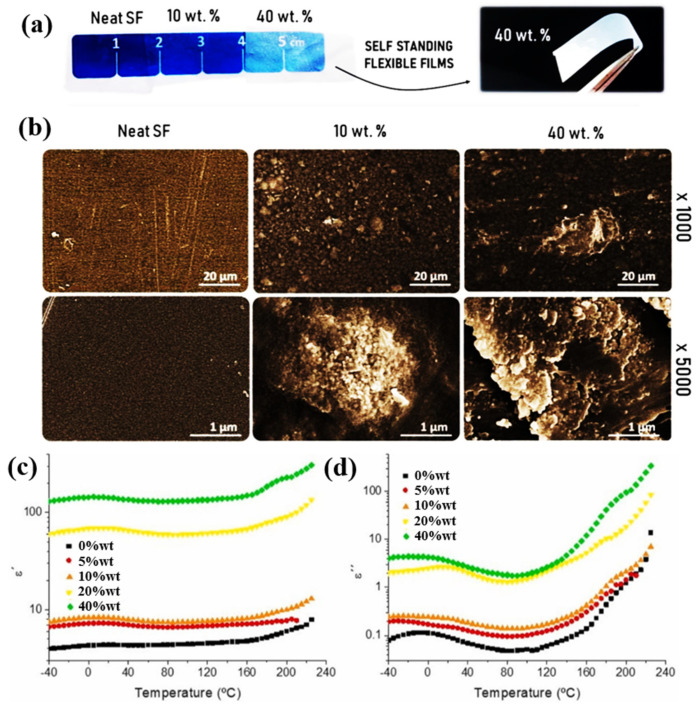
(**a**) Images of flexible silk fibroin/BaTiO_3_ composite films. (**b**) Cross-sectional morphology of silk fibroin films, 10 wt% BaTiO_3_/silk fibroin, and 40 wt% BaTiO_3_/silk fibroin composites. Variation of *ε*′ (**c**) and *ε*″ (**d**) with temperature for BaTiO_3_/silk fibroin composites at 1 KHz. Adapted with permission [86]. Copyright 2021, Elsevier.

Ceramic materials have excellent dielectric properties, but they suffer from poor processing properties due to their low impact resistance and high brittleness, which limit their use in electronics [82]. Therefore, ceramic fillers are commonly applied to prepare high dielectric composites, which own processing and mechanical properties of the polymer matrix while possessing excellent dielectric permittivity of fillers [83,84,85]. However, to improve the dielectric permittivity of composites, a high loading of ceramic fillers is often necessary and the poor compatibility of ceramic fillers with polymers often introduces a large number of defects and voids when the loading is high, which will directly increase dielectric loss and greatly affect mechanical properties of composites. The ceramic filler has a great enhancement of the dielectric permittivity of the composite. Costa et al. used BaTiO_3_ as fillers in a silk fibroin matrix to prepare bio-based composites with high dielectric properties (Figure 6) [86]. The distribution of BaTiO_3_ particles in silk fibroin are relatively uniform. The dielectric permittivity of the composites improved from 4 to 141 at 1 KHZ for BaTiO_3_ addition of 40 wt%. While the dielectric loss also increased from 0.1 to 10, this seriously affects the practical application since high dielectric loss means lot of heat will be generated during use. BaTiO_3_ has very high polarizability, which can greatly improve the dielectric constant of composites. However, as a ceramic filler, the dielectric loss will also greatly increase, which will seriously affect the practical application of polymer high dielectric composites.

**Figure 7:**
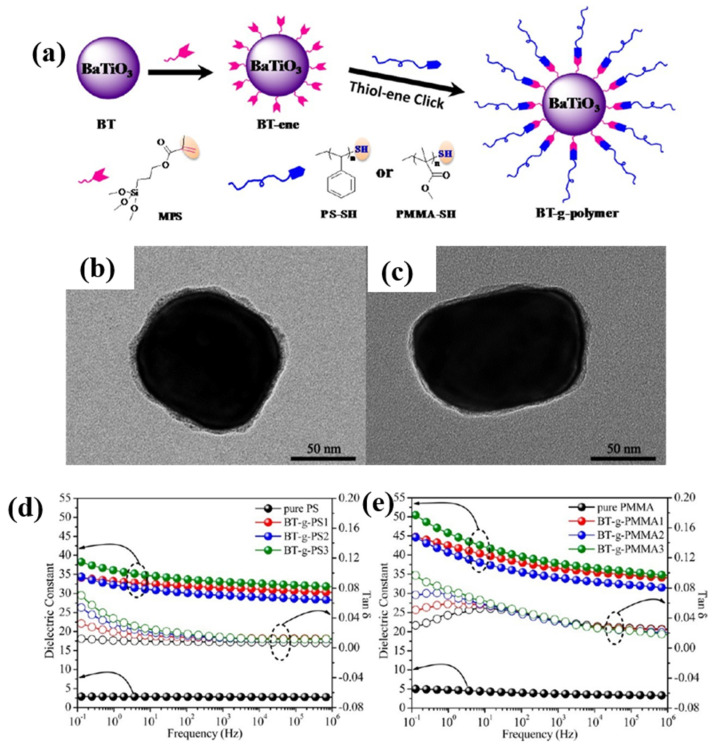
(**a**) Preparation of core-shell structured polymer@BaTiO_3_ Nanoparticles. (**b**) PS@BaTiO_3_ nanoparticle and (**c**) PMMA@BaTiO_3_ at high magnification. Dielectric properties of PS@BaTiO_3_ (**d**) and PMMA@BaTiO_3_ (**e**) composites. Adapted with permission [90]. Copyright 2014, American Chemical Society.

To address the high dielectric loss and poor compatibility of ceramic fillers, many researchers have explored strategies to increase the interaction between a ceramic filler and the matrix [87,88,89]. Yang et al. developed a method of preparing vinylated BaTiO_3_ [90]. Both polystyrene modified BaTiO_3_ (PS@BaTiO_3_) and poly (methyl methacrylate) modified BaTiO_3_ (PMMA@BaTiO_3_) are investigated systematically, as shown in Figure 7. BaTiO_3_ was modified by polymer and the dielectric loss remained very low (<0.04) when the dielectric permittivity was increased above 30 at 100 Hz. BaTiO_3_ often has uneven dispersion phenomenon when used as filler, which is one of the reasons for the high dielectric loss of composites with BaTiO_3_ as filler. Therefore, modifying BaTiO_3_ with polymer materials can improve the compatibility with the matrix and achieve the purpose of suppressing the dielectric loss.

### 3.3. Application of Polymer Fillers in Dielectric Composites

Some polar polymers cannot be used as a matrix for high dielectric polymer composites directly, but they are effective as filler to increase the dielectric properties of the composite due to the very high content of polar groups. For instance, both polyaniline (PANI) and cellulose nanocrystals (CNCs) as fillers can effectively improve the dielectric properties of composites [91,92,93].

**Figure 8:**
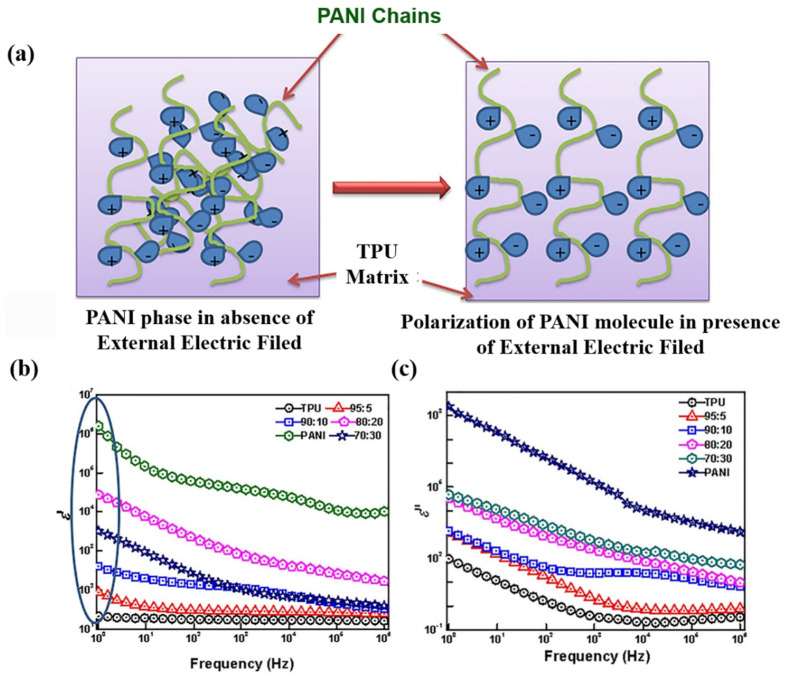
(**a**) Polarization of TPU/PANI composites under electric fields. The effect of PANI loading on *ε*′ (**b**) and *ε*″ (**c**). Adapted with permission [94]. Copyright 2020, Springer Nature.

Polyaniline is a special conductive polymer material. As a filler, it has good compatibility with the matrix and can also improve the dielectric permittivity of composites. Dash et al. has successfully enhanced the dielectric properties of thermoplastic polyurethane (TPU) using polyaniline (PANI) as a filler [94]. As shown in Figure 8, both *ε*′ and *ε*″ of the composites increase with the addition of PANI. When the amount of PANI is less than 15 wt%, the break strength of the composite increased. PANI as a special conductive polymer can not only improve the dielectric constant of the composites, but also PANI has excellent compatibility with the polymer matrix when added to the polymer at a certain content and has a low impact on the dielectric loss of the composite (as shown in Figure 8c). Wu et al. prepared PANI/PDMS composites by orienting PANI fillers in a polydimethylsiloxane (PDMS) matrix through AC electric fields [91]. When the PANI addition was 10%, the dielectric permittivity of PANI/PDMS (random) increased by 1.72 at 100 Hz and that of PANI/PDMS (aligned) increased by 96.02 at 100 Hz. In addition to being used as a filler, many researchers use PANI to modify the filler to prepare composite materials with excellent dielectric properties. Zhang et al. prepared composites with excellent dielectric properties by embedding polyaniline modified BaTiO_3_ (BT@PANI) as fillers into polyvinylidene cohexafluoropropylene [P(VDF-HFP)] [95]. With 20 vol% of BT@PANI, the dielectric permittivity of the composite can reach 99.1 at 1 KHz, which is 83 higher than that of 20 vol% BT (16.1) and 88.8 higher than that of the original P(VDF-HDP) (10.3). In the study of Rahnamol A. M. et al., PANI and GO hybrid materials were used as fillers to increase the dielectric properties of an epoxy resin matrix [96]. In the work of many researchers, PANI not only provides excellent dielectric properties for composite materials but also has better compatibility with the matrix and thus results in better mechanical properties. 

**Figure 9:**
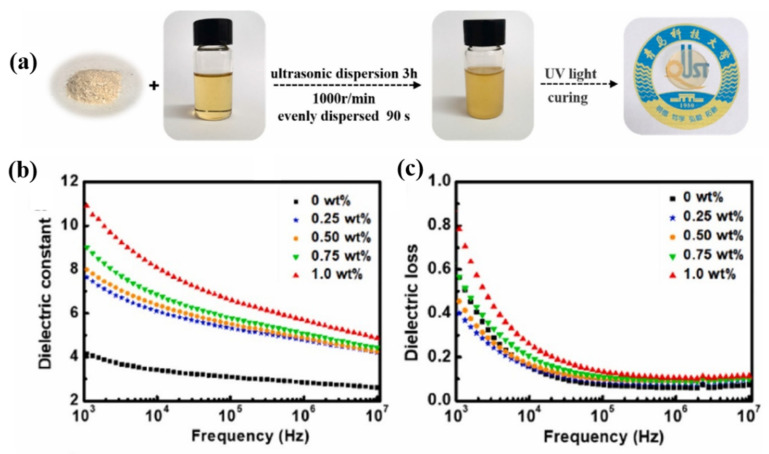
(**a**) preparation of MMPR/CNCs-MAA. Dielectric permittivity (**b**) and dielectric loss (**c**) of MMPR/CNCs-MAA. Adapted with permission [93]. Copyright 2022, Elsevier.

In our earlier study, CNCs were modified by methacrylic acid and then dispersed into UV curable resin methacrylate malate photocurable resin (MMPR) to prepare high dielectric CNCs-MAA/MMPR composites [93]. The modified CNCs can dispersed in the resin well and enhance the dielectric properties (Figure 9). With the addition of 1.0 wt% CNCs-MAA, the dielectric permittivity increased from 4.0 to 10.9 at 1 KHz, while the dielectric loss was only improved by 0.22. The influence of CNCs on the dielectric properties of composites mainly includes polar groups and the interface effect. The modified CNCs will have better dispersion in the matrix, but if the grafted polymer chain is too long, the interface effect will be affected, and the dielectric constant will be less improved. Asma Khouaja et al. used cellulose to enhance the dielectric permittivity of high density polyethylene (HDPE) [97]. With a 50% addition of cellulose, the dielectric permittivity of HDPE at 10^6^ Hz increased from 1.3 to 2.2. This shows that it is feasible to enhance the dielectric properties of the composites by polymer fillers, such as CNCs, PANI, which provides a new insight for research on all-organic high dielectric polymer composites [98,99].

## 4. Influence of Structural Design to Dielectric Properties of Polymer Composites

The dielectric properties of composites are influenced not only by the matrix and filler but also by the distribution of the filler in the matrix. The orientation of the fillers in a certain direction can improve the dielectric permittivity in this direction to a large extent [46,55,57]. Shear force, magnetic fields, and electric fields have been developed to orient fillers in a specific direction to prepare dielectric materials of outstanding dielectric properties [100,101,102,103].

**Figure 10:**
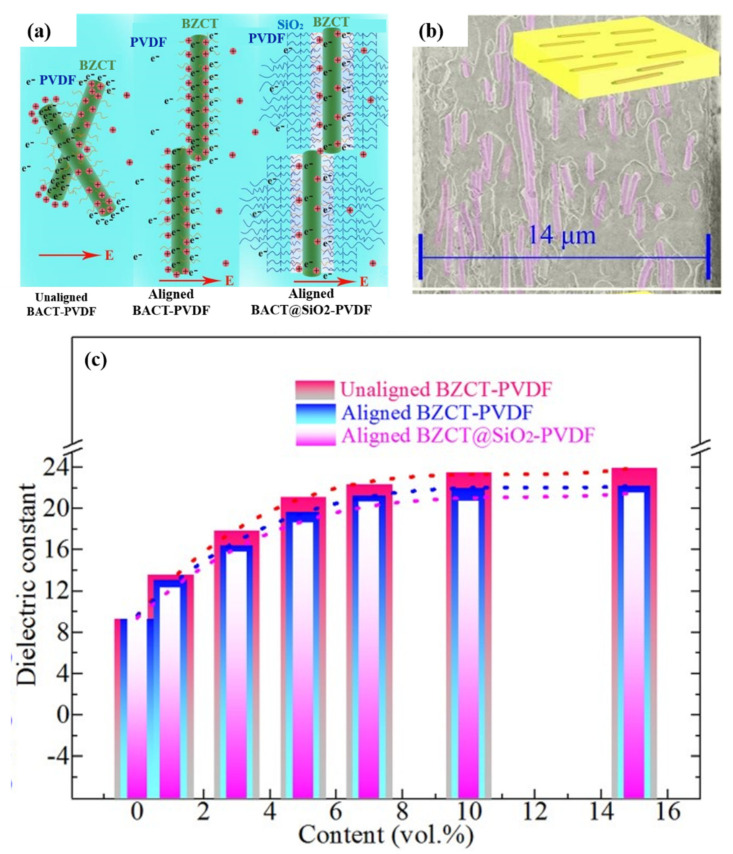
(**a**) Schematic illustration of polarization behavior of BZCT and BZCT@SiO_2_ nanofibers. (**b**) SEM image of oriented structure. (**c**) Dielectric permittivity of composites after orientation at 10 Hz. Adapted with permission [104]. Copyright 2019, Elsevier.

Under tensile or shear forces, fillers in the polymer matrix can be aligned in the direction of the force. Zhang et al. oriented 0.5Ba(Zr_0.2_Ti_0.8_)O_3_-0.5(Ba_0.7_Ca_0.3_)TiO_3_ (BZCT) BZCT and BZCT@SiO_2_ nanofibers in PVDF by shear force during electrospinning [104]. Two nanocomposites, BZCT-PVDF and BZCT@SiO_2_-PVDF, were prepared by the hot-pressing method. As shown in Figure 10, the orientation direction of the nanofibers is perpendicular to the direction of the electric field and the orientation structure of fibers in the matrix is obvious. The dielectric permittivity of BZCT-PVDF at 10 Hz decreases from 24 to 22 when the nanofiber content is 15 vol% after orientation. Tang et al. used the uniaxial tensile method to orient lead zirconate titanate nanowires (PZT-NWs) in a thermoplastic elastomer [102]. The orientation of the oriented nanowires was also perpendicular to the electric field. At a frequency of 1 KHz with a PZT-NWs content of 40%, the dielectric permittivity of the oriented composite decreases from 40 to 25. The orientation can increase the amount of filler in a certain direction. If the orientation is wrong, the result will be a decrease in the amount of filler in this direction. Although the orientation of the force field can complete the orientation process smoothly and quickly, the orientation process often requires a large displacement, which greatly limits the orientation direction. Therefore, magnetic field and electric field, which are convenient and simple orientation methods, are favored by more researchers. 

**Figure 11:**
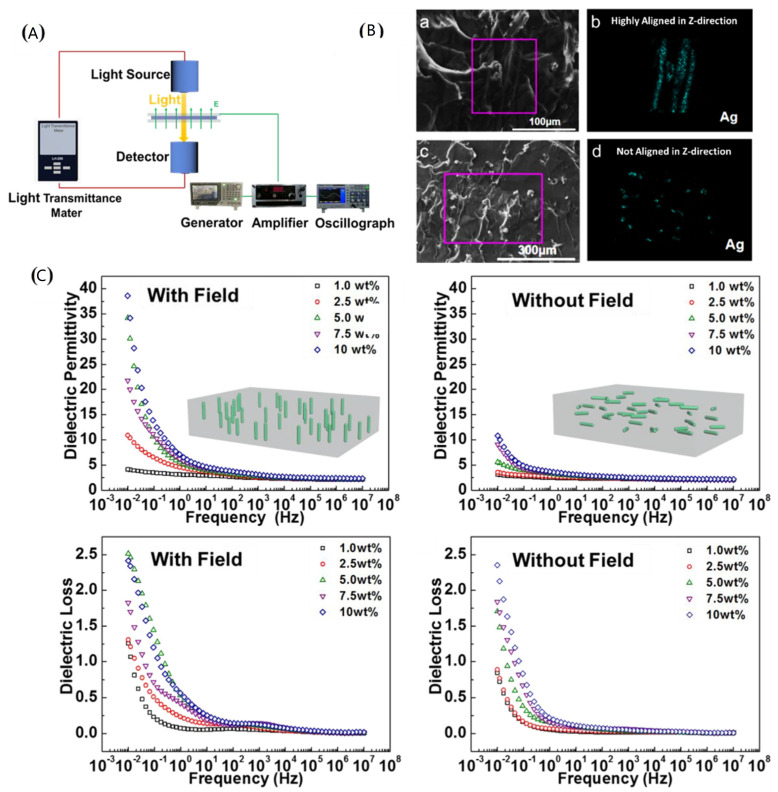
(**A**) Preparation of high dielectric composites by electric fields. (**B**) SEM image of oriented structures. (**C**) Dielectric properties of composites after orientation. Adapted with permission [101]. Copyright 2020, American Chemical Society.

The electric or magnetic fields can rapidly orient the fillers and produce thin films with oriented structures along the direction of electric fields. Chen et al. prepared high dielectric composites with excellent properties by orientations of silver-coated cellulose nanocrystals in silicone rubber by the electric field (Figure 11) [101]. The orientation process could be completed in only 90s. The dielectric permittivity of the oriented composites was significantly improved and the dielectric permittivity after orientation was increased from 13.8 to 38.6 at 10^−2^ Hz for 10 wt% silver plated CNC and the dielectric loss did not increase significantly. The silver-coated cellulose nanocrystals have a higher response rate under the electric field, which makes the cellulose nanocrystals more easily oriented. The orientation process increases the amount of cellulose in the direction of the electric filed, so the dielectric constant increases. Of course, at low filler content, the orientation process has little effect on the dielectric loss and if the content is high, the dielectric loss will rise sharply.

## 5. Regulation Strategies of Dielectric Loss

**Figure 12:**
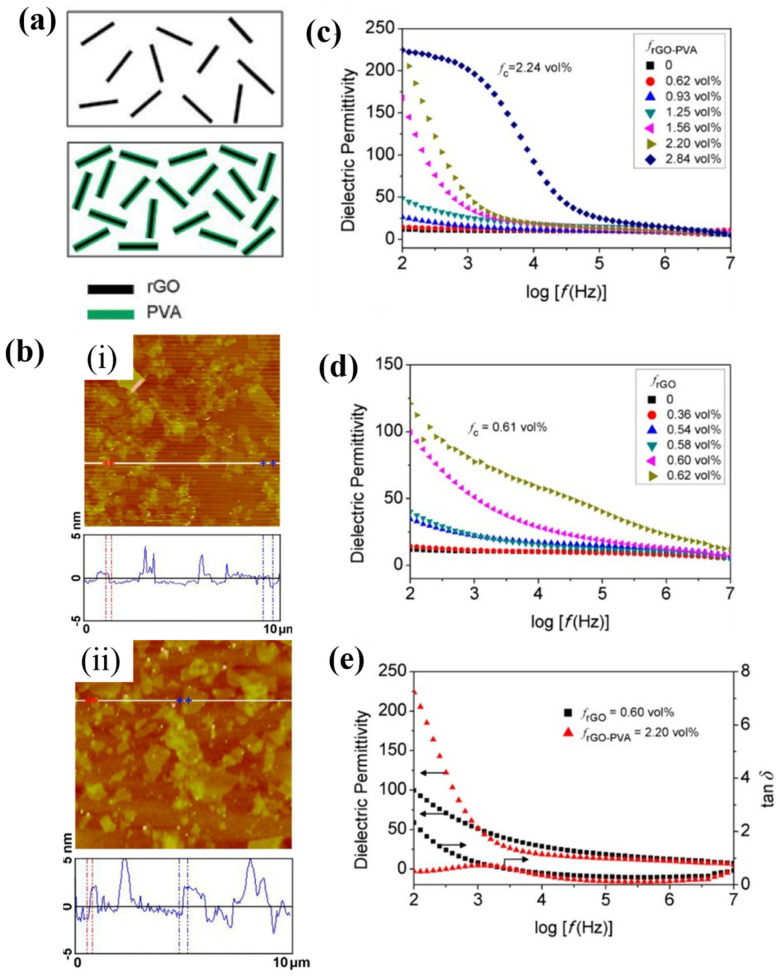
(**a**) Sketches of rGO and rGO-PVA. (**b**) AFM images of (**i**) rGO and (**ii**) rGO-PVA. Dielectric permittivity of (**c**) rGO-PVA/PVDF films and (**d**) rGO/PVDF films. (**e**) Dielectric properties of nanocomposites. Adapted with permission [107]. Copyright 2012, American Chemical Society.

High dielectric loss can seriously affect the practical application of high dielectric polymer composites. Dielectric loss represents the heat generated by the dielectric when it consumes part of the electric energy in the alternating electric field, which is mainly affected by polarization loss and conductivity loss. Polarization loss can hardly be avoided, while the conductivity loss caused dielectric loss can be effectively manipulated. Conductivity loss is caused by the current flow generated inside of the material. Therefore, conductance loss can be reduced by blocking the conductive pathways [105,106]. The key to reducing dielectric loss is to prevent the fillers from contacting each other to form conductive pathways, since the polymer matrix is not conductive. As Figure 12 shows, Wang et al. prepared rGO-PVA by polyvinyl alcohol modification of reduced grapheme oxide (rGO) to block the mutual contact between rGO in the PVDF matrix [107]. The dielectric permittivity of 2.2 vol% rGO-PVA/PVDF composite is up to 230 at 100 Hz and the dielectric loss remains low. While the dielectric loss of 2.2 vol% rGO/PVDF is as high as 50, which is unacceptable for practical energy storage use. It can be concluded that the coated insulation layers can effectively block the conductive pathways to suppress the rise of dielectric loss. The fillers are evenly dispersed into the matrix after being coated with insulation coating. In this way, the fillers can not only improve the dielectric constant of the composite but also prevent the filler from contacting each other, so as to achieve the purpose of inhibiting the dielectric loss. Therefore, in the report of wang et al., the dielectric constant of composite materials is increased while the dielectric loss is lower. Similarly, Yang et al. prepared PS/BaTiO_3_ (BT-PS) by modifying BaTiO_3_ with polystyrene (PS) and prepared composites with BT-PS as the filler and PS as the matrix [108]. The PS shell effectively inhibits the enhancement of dielectric loss. With addition of 47.69 vol% BaTiO_3_, the dielectric loss of PS/BaTiO_3_ composites at 1 KHZ was increased by as low as 0.005.

**Figure 13:**
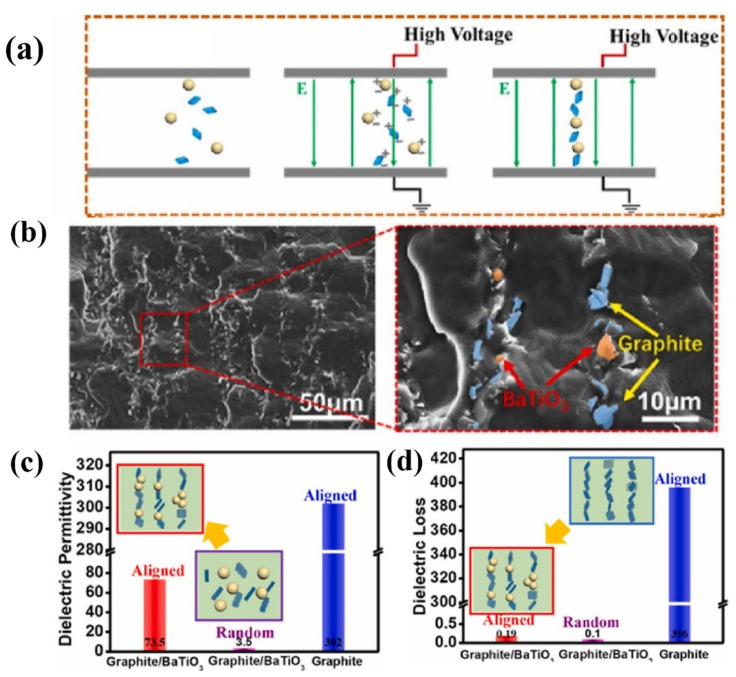
(**a**) A scheme of perparation of aligned copmposites. (**b**) Cross-sectional SEM images of the composites. Dielectric permittivity (**c**) and dielectric loss (**d**) of composite films. Adapted with permission [109]. Copyright 2021, Elsevier.

In addition to the modification of fillers with polymers, incorporating insulative particles into potential conductive pathways is also an eye-catching approach. To prevent the formation of a conductive pathway, the method of using barium titanate (BaTiO_3)_ particles as a barrier to block the formation of a conductive pathway was ingeniously proposed and achieved quite good performance (Figure 13) [109]. As shown in Figure 13c,d, when the graphite content was 2.5 wt%, the dielectric loss increased to as high as 396 for aligned composites; however, the dielectric loss could be reduced to 0.19 by blocking the formation of the conductive pathway by adding 5 wt% insulating BaTiO_3_. Note that the dielectric permittivity remained at a high value (73.5) after introducing insulating BaTiO_3_. BaTiO_3_ blocks the mutual contact of PANI and reduces the formation of conductive pathways, which can significantly reduce the conductivity loss in the composite, so the dielectric loss of the composite is still low after orientation.

## 6. Summary and Outlook

In summary, the influences of the matrix, fillers, and filler arrangement on dielectric properties are systematically and comprehensively summarized, and the regulation strategies of dielectric loss are introduced as well. The effect of the polarity of the matrix on dielectric properties and the influences of conductive fillers, ceramic fillers, and polar polymer fillers on the dielectric properties of composites are described. The advantages and disadvantages of different type fillers are listed. The influence of dielectric properties of oriented structures and various orientation methods, including electric fields, shear force, and magnetic fields, are also introduced. Moreover, the methods to inhibit the increase of dielectric loss, including coating insulation and introducing insulation particles, are reviewed.

While considerable progress has been made in the research of high dielectric composites in recent years, challenges and bottlenecks still exists. Informed research and technologies in high dielectric composites are still limited to the laboratory state. The reported methods to inhibit the rise of dielectric loss are cumbersome, which limits its commercial process. In addition, the preparation of high dielectric composite materials is still dominated by traditional methods, which make it difficult to prepare complex and ingenious structures. 

In the near future, the development of high dielectric polymer composites is no longer limited to the use of only one type of filler in one system. Instead, multiple fillers and multiple structures desiderate to be designed and combined in high dielectric polymer composites to enhance dielectric properties by synergistic effect [109,110]. New strategies of restraining dielectric loss are also urgently needed since the previous methods of coating insulating layer on fillers or introducing insulating fillers into conductive fillers are still difficult to scaleup in practical application. The application of biomaterials is also a new trend of high dielectric composites due to the growing piles of electronic trash. In order to solve the environmental problems caused by electronic trash, more and more biological matrix and bio-based fillers will be applied for the preparation of high dielectric composites. Note here, the long-term stability of the biological matrix and bio-based fillers during electronic environments need to be systematically investigated. Finally, 3D printing is needed in future studies on the preparation of high dielectric polymer composites, which will have positive implications for demonstrating the increasingly complicated electronic design in numerous fields.

## Figures and Tables

**Table 1:** Dielectric properties of various polymers [6].

Polymer Materials	Dielectric Permittivity (1 KHz)	Loss Tangent (1 KHz)	References
Polytetrafluoroethylene (PTFE)	2	0.0001	[25]
Biaxially oriented polypropylene (BOPP)	2.2	0.0002	[26]
Low-Density Polyethylene (LDPE)	2.3	0.003	[25,27]
High-Density Polyethylene (HDPE)	2.3	0.0002–0.0007	[25,27]
Polystyrene (PS)	2.4–2.7	0.008	[28]
Polydimethyl siloxane (PDMS)	2.6	0.01	[25,29]
Polycarbonate (PC)	3.0	0.0015	[28]
Polyvinyl chloride (PVC)	3.4	0.018	[30]
Polyimide (PI)	3.5	0.04	[31]
Polyethylene glycol terephthalate (PET)	3.6	0.01	[25,32]
Poly(ether-ether-ketone) (PEEK)	4.0	0.009 (100 KHz)	[25,33,34]
Epoxy	4.5	0.015	[25,28,35,36]
Polymethyl methacrylate (PMMA)	4.5	0.05	[25,37,38,39]
Polyurethane (PU)	4.6	0.02	[40]
Polyvinylidene difluoride (PVDF)	10	0.04	[36]
Polyvinyl alcohol (PVA)	12	0.3	[41,42]

**Table 2:** Common high dielectric fillers and their advantages and disadvantages.

Type of Filler	Common Materials	Advantages	Disadvantages	References
Conductive fillers	Ag, Au, CNTs, Graphene, etc.	High dielectric	Difficult to disperse; Poor compatibility	[44,45,46,47]
Ceramic fillers	BaSrTiO_3_, BaTiO_3_, CaCuTiO_3_, etc.	High dielectric	High dielectric loss; Poor compatibility	[48,49,50]
Polar polymers	CNCs, PAN, etc.	Good compatibility	Limited increase of dielectric permittivity	[51,52,53,54,55]

## Data Availability

Not applicable.

## References

[1] Mendes-Felipe C., Barbosa J., Gonçalves S., Pereira N., Costa C., Vilas-Vilela J., Lanceros-Mendez S. (2020). High dielectric constant UV curable polyurethane acrylate/indium tin oxide composites for capacitive sensing. Compos. Sci. Technol..

[2] Asiri A.M. (2019). Inamuddin. Ionic Polymer Metal Composites for Sensors and Actuators.

[3] Zhang Y., Lin B., Sun Y., Han P., Wang J., Ding X., Zhang X., Yang H. (2016). MoO2@ Cu@ C Composites Prepared by Using Polyoxometalates@ Metal-Organic Frameworks as Template for All-Solid-State Flexible Supercapacitor. Electrochim. Acta.

[4] Ma T., Zhao Y., Ruan K., Liu X., Zhang J., Guo Y., Yang X., Kong J., Gu J. (2019). Highly Thermal Conductivities, Excellent Mechanical Robustness and Flexibility, and Outstanding Thermal Stabilities of Aramid Nanofiber Composite Papers with Nacre-Mimetic Layered Structures. ACS Appl. Mater. Interfaces.

[5] Zhang D.-L., Liu S.-N., Cai H.-W., Feng Q.-K., Zhong S.-L., Zha J.-W., Dang Z.-M. (2020). Enhanced thermal conductivity and dielectric properties in electrostatic self-assembly 3D pBN@nCNTs fillers loaded in epoxy resin composites. J. Materiomics.

[6] Thakur V.K., Gupta R.K. (2016). Recent Progress on Ferroelectric Polymer-Based Nanocomposites for High Energy Density Capacitors: Synthesis, Dielectric Properties, and Future Aspects. Chem. Rev..

[7] Wang B., Huang W., Chi L., Al-Hashimi M., Marks T.J., Facchetti A. (2018). High-*k* Gate Dielectrics for Emerging Flexible and Stretchable Electronics. Chem. Rev..

[8] Li Y., Krentz T.M., Wang L., Benicewicz B.C., Schadler L.S. (2014). Ligand Engineering of Polymer Nanocomposites: From the Simple to the Complex. ACS Appl. Mater. Interfaces.

[9] Yuan J.-K., Yao S.-H., Dang Z.-M., Sylvestre A., Genestoux M., Bai J. (2011). Giant Dielectric Permittivity Nanocomposites: Realizing True Potential of Pristine Carbon Nanotubes in Polyvinylidene Fluoride Matrix through an Enhanced Interfacial Interaction. J. Phys. Chem. C.

[10] Dang Z.-M., Yuan J.-K., Zha J.-W., Zhou T., Li S.-T., Hu G.-H. (2012). Fundamentals, processes and applications of high-permittivity polymer–matrix composites. Prog. Mater. Sci..

[11] Fan P., Wang L., Yang J., Chen F., Zhong M. (2012). Graphene/poly(vinylidene fluoride) composites with high dielectric constant and low percolation threshold. Nanotechnology.

[12] Feng Q.-K., Zhong S.-L., Pei J.-Y., Zhao Y., Zhang D.-L., Liu D.-F., Zhang Y.-X., Dang Z.-M. (2021). Recent Progress and Future Prospects on All-Organic Polymer Dielectrics for Energy Storage Capacitors. Chem. Rev..

[13] Patel P.K., Rani J., Yadav K. (2020). Effective strategies for reduced dielectric loss in ceramic/polymer nanocomposite film. Ceram. Int..

[14] Aepuru R., Mondal S., Ghorai N., Kumar V., Panda H.S., Ghosh H.N. (2018). Exploring the Carrier Dynamics in Zinc Oxide–Metal Halide-Based Perovskite Nanostructures: Toward Reduced Dielectric Loss and Improved Photocurrent. J. Phys. Chem. C.

[15] Li Q., Cheng J., Chen J. (2017). Reduced dielectric loss and enhanced piezoelectric properties of Mn modified 0.71 BiFeO_3_–0.29 BaTiO_3_ ceramics sintered under oxygen atmosphere. J. Mater. Sci. Mater. Electron..

[16] Deng Y.-Y., Zhou D.-L., Han D., Zhang Q., Chen F., Fu Q. (2020). Fluoride ion encapsulated polyhedral oligomeric silsesquioxane: A novel filler for polymer nanocomposites with enhanced dielectric constant and reduced dielectric loss. Compos. Sci. Technol..

[17] Hu Z., Wang Y., Liu X., Wang Q., Cui X., Jin S., Yang B., Xia Y., Huang S., Qiang Z. (2022). Rational design of POSS containing low dielectric resin for SLA printing electronic circuit plate composites. Compos. Sci. Technol..

[18] Zhang Z., Tian D., Niu Z., Zhou Y., Hou X., Ma X. (2021). Enhanced toughness and lowered dielectric loss of reactive POSS modified bismaleimide resin as well as the silica fiber reinforced composites. Polym. Compos..

[19] Zhu L. (2014). Exploring Strategies for High Dielectric Constant and Low Loss Polymer Dielectrics. J. Phys. Chem. Lett..

[20] Zhu L., Wang Q. (2012). Novel Ferroelectric Polymers for High Energy Density and Low Loss Dielectrics. Macromolecules.

[21] He D., Wang Y., Song S., Liu S., Luo Y., Deng Y. (2017). Polymer-based nanocomposites employing Bi_2_S_3_@ SiO_2_ nanorods for high dielectric performance: Understanding the role of interfacial polarization in semiconductor-insulator core-shell nanostructure. Compos. Sci. Technol..

[22] Marx P., Wanner A.J., Zhang Z., Jin H., Tsekmes I.-A., Smit J.J., Kern W., Wiesbrock F. (2017). Effect of Interfacial Polarization and Water Absorption on the Dielectric Properties of Epoxy-Nanocomposites. Polymers.

[23] Wang Q., Wu C., LaChance A.M., Zhou J., Gao Y., Zhang Y., Sun L., Cao Y., Liang X. (2022). Interfacial polarization suppression of P(VDF-HFP) film through 2D montmorillonite nanosheets coating. Prog. Org. Coat..

[24] Zhang X., Ye H., Xu L. (2022). Exploring the interfacial polarization in poly(vinylidene fluoride-chlorotrifluoroethylene) dielectric film with regulated surface conductivity of C@BT particles. Appl. Surf. Sci..

[25] Huang X., Jiang P., Tanaka T. (2011). A review of dielectric polymer composites with high thermal conductivity. IEEE Electr. Insul. Mag..

[26] Han C., Zhang X., Chen D., Ma Y., Zhao C., Yang W. (2020). Enhanced dielectric properties of sandwich-structured biaxially oriented polypropylene by grafting hyper-branched aromatic polyamide as surface layers. J. Appl. Polym. Sci..

[27] Mohamed A.T. (2015). Experimental enhancement for dielectric strength of polyethylene insulation materials using cost-fewer nanoparticles. Int. J. Electr. Power Energy Syst..

[28] Barber P., Balasubramanian S., Anguchamy Y., Gong S., Wibowo A., Gao H., Ploehn H.J., Zur Loye H.-C. (2009). Polymer Composite and Nanocomposite Dielectric Materials for Pulse Power Energy Storage. Materials.

[29] Molberg M., Crespy D., Rupper P., Nüesch F., Månson J.-A.E., Löwe C., Opris D.M. (2010). High Breakdown Field Dielectric Elastomer Actuators Using Encapsulated Polyaniline as High Dielectric Constant Filler. Adv. Funct. Mater..

[30] Abouhaswa A.S., Taha T.A. (2019). Tailoring the optical and dielectric properties of PVC/CuO nanocomposites. Polym. Bull..

[31] Pan J., Li K., Chuayprakong S., Hsu T., Wang Q. (2010). High-Temperature Poly(phthalazinone ether ketone) Thin Films for Dielectric Energy Storage. ACS Appl. Mater. Interfaces.

[32] Coburn J.C., Boyd R.H. (1986). Dielectric relaxation in poly(ethylene terephthalate). Macromolecules.

[33] Goyal R.K., Madav V.V., Pakankar P.R., Butee S.P. (2011). Fabrication and properties of novel polyetheretherketone/barium ti-tanate composites with low dielectric loss. J. Electron. Mater..

[34] Pan J., Li K., Li J., Hsu T., Wang Q. (2009). Dielectric characteristics of poly(ether ketone ketone) for high temperature capacitive energy storage. Appl. Phys. Lett..

[35] Fang L., Wu C., Qian R., Xie L., Yang K., Jiang P. (2014). Nano–micro structure of functionalized boron nitride and aluminum oxide for epoxy composites with enhanced thermal conductivity and breakdown strength. RSC Adv..

[36] Song Y., Shen Y., Liu H., Lin Y., Li M., Nan C.-W. (2012). Improving the dielectric constants and breakdown strength of polymer composites: Effects of the shape of the BaTiO_3_ nanoinclusions, surface modification and polymer matrix. J. Mater. Chem..

[37] Paniagua S.A., Kim Y., Henry K., Kumar R., Perry J.W., Marder S.R. (2014). Surface-Initiated Polymerization from Barium Titanate Nanoparticles for Hybrid Dielectric Capacitors. ACS Appl. Mater. Interfaces.

[38] Xie L., Huang X., Huang Y., Yang K., Jiang P. (2013). Core@Double-Shell Structured BaTiO_3_–Polymer Nanocomposites with High Dielectric Constant and Low Dielectric Loss for Energy Storage Application. J. Phys. Chem. C.

[39] Gross S., Camozzo D., DI Noto V., Armelao L., Tondello E. (2007). PMMA: A key macromolecular component for dielectric low-κ hybrid inorganic–organic polymer films. Eur. Polym. J..

[40] Chen T., Zhao Y., Pan L., Lin M. (2015). Insight into effect of hydrothermal preparation process of nanofillers on dielectric, creep and electromechanical performance of polyurethane dielectric elastomer/reduced graphene oxide composites. J. Mater. Sci. Mater. Electron..

[41] Das A., Sinha S., Mukherjee A., Meikap A. (2015). Enhanced dielectric properties in polyvinyl alcohol—Multiwall carbon nanotube composites. Mater. Chem. Phys..

[42] Tuncer E., Sauers I., James D.R., Ellis A.R., Duckworth R.C. (2008). Nanodielectric system for cryogenic applications: Barium titanate filled polyvinyl alcohol. IEEE Trans. Dielectr. Electr. Insul..

[43] Shen Y., Lin Y.H., Nan C.-W. (2007). Interfacial Effect on Dielectric Properties of Polymer Nanocomposites Filled with Core/Shell-Structured Particles. Adv. Funct. Mater..

[44] Xie X., Yang C., Qi X.-D., Yang J.-H., Zhou Z.-W., Wang Y. (2019). Constructing polymeric interlayer with dual effects toward high dielectric constant and low dielectric loss. Chem. Eng. J..

[45] Gong Y., Zhou W., Sui X., Kou Y., Xu L., Duan Y., Chen F., Li Y., Liu X., Cai H. (2018). Core-shell structured Al/PVDF nanocomposites with high dielectric permittivity but low loss and enhanced thermal conductivity. Polym. Eng. Sci..

[46] Zhou W., Chen Q., Sui X., Dong L., Wang Z. (2015). Enhanced thermal conductivity and dielectric properties of Al/β-SiCw/PVDF composites. Compos. Part A Appl. Sci. Manuf..

[47] Salehiyan R., Nofar M., Makwakwa D., Ray S.S. (2020). Shear-Induced Carbon Nanotube Migration and Morphological Development in Polylactide/Poly(vinylidene fluoride) Blend Nanocomposites and Their Impact on Dielectric Constants and Rheological Properties. J. Phys. Chem. C.

[48] Zhang L., Shan X., Bass P., Tong Y., Rolin T.D., Hill C.W., Brewer J.C., Tucker D.S., Cheng Z.-Y. (2016). Process and Microstructure to Achieve Ultra-high Dielectric Constant in Ceramic-Polymer Composites. Sci. Rep..

[49] Dai Z.-H., Li T., Gao Y., Xu J., He J., Weng Y.-X., Guo B.-H. (2018). Achieving high dielectric permittivity, high breakdown strength and high efficiency by cross-linking of poly(vinylidene fluoride)/BaTiO_3_ nanocomposites. Compos. Sci. Technol..

[50] Liu W., Lee S.W., Lin D., Shi F., Wang S., Sendek A.D., Cui Y. (2017). Enhancing ionic conductivity in composite polymer electrolytes with well-aligned ceramic nanowires. Nat. Energy.

[51] Zhang Q.M., Li H., Poh M., Xia F., Cheng Z.-Y., Xu H., Huang C. (2002). An all-organic composite actuator material with a high dielectric constant. Nature.

[52] Ltaief A.O., Ghorbel N., Benhamou K., Arous M., Kaddami H., Kallel A. (2021). Impact of cellulose nanocrystals reinforcement on molecular dynamics and dielectric properties of PCL-based polyurethane. Polym. Compos..

[53] Ladhar A., Ben Mabrouk A., Arous M., Boufi S., Kallel A. (2017). Dielectric properties of nanocomposites based on cellulose nanocrystals (CNCs) and poly(styrene-co-2-ethyl hexylacrylate) copolymer. Polymer.

[54] Ma L., Liu R., Niu H., Zhao M., Huang Y. (2016). Flexible and freestanding electrode based on polypyrrole/graphene/bacterial cellulose paper for supercapacitor. Compos. Sci. Technol..

[55] Guo Y., Chen Y., Wang E., Cakmak M. (2017). Roll-to-Roll Continuous Manufacturing Multifunctional Nanocomposites by Electric-Field-Assisted “Z” Direction Alignment of Graphite Flakes in Poly(dimethylsiloxane). ACS Appl. Mater. Interfaces.

[56] Martin J.J., Fiore B.E., Erb R.M. (2015). Designing bioinspired composite reinforcement architectures via 3D magnetic printing. Nat. Commun..

[57] Xu S., Liu D., Zhang Q., Fu Q. (2017). Electric field-induced alignment of nanofibrillated cellulose in thermoplastic polyurethane matrix. Compos. Sci. Technol..

[58] Kadimi A., Benhamou K., Ounaies Z., Magnin A., Dufresne A., Kaddami H., Raihane M. (2014). Electric Field Alignment of Nanofibrillated Cellulose (NFC) in Silicone Oil: Impact on Electrical Properties. ACS Appl. Mater. Interfaces.

[59] Tsyganov A., Vikulova M., Artyukhov D., Bainyashev A., Goffman V., Gorokhovsky A., Boychenko E., Burmistrov I., Gorshkov N. (2022). Permittivity and Dielectric Loss Balance of PVDF/K_1.6_Fe_1.6_Ti_6.4_O_16_/MWCNT Three-Phase Composites. Polymers.

[60] Deeba F., Gupta A.K., Kulshrestha V., Bafna M., Jain A. (2022). Analysing the dielectric properties of ZnO doped PVDF/PMMA blend composite. J. Mater. Sci. Mater. Electron..

[61] Celebi H., Duran S., Dogan A. (2022). The effect of core-shell BaTiO_3_@ SiO2 on the mechanical and dielectric properties of PVDF composites. Polym.-Plast. Technol. Mater..

[62] Silakaew K., Swatsitang E., Thongbai P. (2022). Novel polymer composites of RuO_2_@ nBaTiO_3_/PVDF with a high dielectric constant. Ceram. Int..

[63] Jing L., Li W., Gao C., Li M., Fei W. (2022). Excellent energy storage properties achieved in PVDF-based composites by designing the lamellar-structured fillers. Compos. Sci. Technol..

[64] Zhang T., Sun Q., Kang F., Wang Z., Xue R., Wang J., Zhang L. (2022). Sandwich-structured polymer dielectric composite films for improving breakdown strength and energy density at high temperature. Compos. Sci. Technol..

[65] Li Z., Shen Z., Yang X., Zhu X., Zhou Y., Dong L., Xiong C., Wang Q. (2020). Ultrahigh charge-discharge efficiency and enhanced energy density of the sandwiched polymer nanocomposites with poly(methyl methacrylate) layer. Compos. Sci. Technol..

[66] Liao Y., Weng Y., Wang J., Zhou H., Lin J., He S. (2019). Silicone Rubber Composites with High Breakdown Strength and Low Dielectric Loss Based on Polydopamine Coated Mica. Polymers.

[67] Anjeline C.J., Mali D., Lakshminarasimhan N. (2021). High dielectric constant of NiFe_2_O_4_–LaFeO_3_ nanocomposite: Interfacial conduction and dielectric loss. Ceram. Int..

[68] Wang H., Wang Q., Zhang Q., Yang H., Dong J., Cheng J., Tong J., Wen J. (2021). High thermal conductive composite with low dielectric constant and dielectric loss accomplished through flower-like Al2O3 coated BNNs for advanced circuit substrate applications. Compos. Sci. Technol..

[69] Huang Z.-X., Zhao M.-L., Zhang G.-Z., Song J., Qu J.-P. (2021). Controlled localizing multi-wall carbon nanotubes in polyvinylidene fluoride/acrylonitrile butadiene styrene blends to achieve balanced dielectric constant and dielectric loss. Compos. Sci. Technol..

[70] Ding X., Pan Z., Zhang Y., Shi S., Cheng Y., Chen H., Li Z., Fan X., Liu J., Yu J. (2022). Regulation of Interfacial Polarization and Local Electric Field Strength Achieved Highly Energy Storage Performance in Polyetherimide Nanocomposites at Elevated Temperature via 2D Hybrid Structure. Adv. Mater. Interfaces.

[71] Bouiri E.M., Farhan R., Chakhchaoui N., Oumghar K., Denktas C., Eddiai A., Meddad M., Mazroui M., Cherkaoui O., Omari L.E.H. (2022). Improving dielectric properties of composites thin films with polylactic acid and PZT microparticles induced by interfacial polarization. Eur. Phys. J. Appl. Phys..

[72] Bronnikov S., Kostromin S., Asandulesa M., Pankin D., Podshivalov A. (2020). Interfacial interactions and interfacial polarization in polyazomethine/MWCNTs nanocomposites. Compos. Sci. Technol..

[73] Kuang X., Liu Z., Zhu H. (2013). Dielectric properties of Ag@ C/PVDF composites. J. Appl. Polym. Sci..

[74] Tuichai W., Kum-Onsa P., Danwittayakul S., Manyam J., Harnchana V., Thongbai P., Phromviyo N., Chindaprasirt P. (2021). Significantly Enhanced Dielectric Properties of Ag-Deposited (In_1/2_Nb_1/2_)_0.1_Ti_0.9_O_2_/PVDF Polymer Composites. Polymers.

[75] Kaur M., Kumar V., Singh J., Datt J., Sharma R. (2022). Effect of Cu-N co-doping on the dielectric properties of ZnO nanoparticles. Mater. Technol..

[76] Huang A., Liu F., Cui Z., Wang H., Song X., Geng L., Peng X. (2021). Novel PTFE/CNT composite nanofiber membranes with enhanced mechanical, crystalline, conductive, and dielectric properties fabricated by emulsion electrospinning and sintering. Compos. Sci. Technol..

[77] Fan X., Zhang A., Li M., Xu H., Xue J., Ye F., Cheng L. (2020). A reduced graphene oxide/bi-MOF-derived carbon composite as high-performance microwave absorber with tunable dielectric properties. J. Mater. Sci. Mater. Electron..

[78] Zhang L., Yuan S., Chen S., Wang D., Han B.-Z., Dang Z.-M. (2015). Preparation and dielectric properties of core–shell structured Ag@polydopamine/poly(vinylidene fluoride) composites. Compos. Sci. Technol..

[79] Wang Y., Zhu L., Zhou J., Jia B., Jiang Y., Wang J., Wang M., Cheng Y., Wu K. (2018). Dielectric properties and thermal conductivity of epoxy resin composite modified by Zn/ZnO/Al2O3 core–shell particles. Polym. Bull..

[80] Mei B., Qin Y., Agbolaghi S. (2021). A review on supramolecules/nanocomposites based on carbonic precursors and dielectric/conductive polymers and their applications. Mater. Sci. Eng. B.

[81] Chen Y., Lin B., Zhang X., Wang J., Lai C., Sun Y., Liu Y., Yang H. (2014). Enhanced dielectric properties of amino-modified-CNT/polyimide composite films with a sandwich structure. J. Mater. Chem. A.

[82] Wang J. (2022). High-Performance Dielectric Ceramic for Energy Storage Capacitors. Coatings.

[83] Feng Y., Li W.L., Hou Y.F., Yu Y., Cao W.P., Zhang T.D., Fei W.D. (2014). Enhanced dielectric properties of PVDF-HFP/BaTiO_3_-nanowire composites induced by interfacial polarization and wire-shape. J. Mater. Chem. C.

[84] Pan Z., Yao L., Zhai J., Shen B., Wang H. (2017). Significantly improved dielectric properties and energy density of polymer nanocomposites via small loaded of BaTiO_3_ nanotubes. Compos. Sci. Technol..

[85] Ding C., Yu S., Tang X., Liu Z., Luo H., Zhang Y., Zhang D., Chen S. (2022). The design and preparation of high-performance ABS-based dielectric composites via introducing core-shell polar polymers@ BaTiO_3_ nanoparticles. Compos. Part A Appl. Sci. Manuf..

[86] Costa C., Reizabal A., i Serra R.S., Balado A.A., Pérez-Álvarez L., Ribelles J.G., Vilas-Vilela J., Lanceros-Méndez S. (2021). Broadband dielectric response of silk Fibroin/BaTiO_3_ composites: Influence of nanoparticle size and concentration. Compos. Sci. Technol..

[87] Chen J., Huang F., Zhang C., Meng F., Cao L., Lin H. (2022). Enhanced energy storage density in poly(vinylidene fluoride-hexafluoropropylene) nanocomposites by filling with core-shell structured BaTiO_3_@ MgO nanoparticals. J. Energy Storage.

[88] Chen J., Zhou C., Cai W., Huang F., Zhang C., Cao L., Meng F. (2022). Pluronic F127-modified BaTiO_3_ for ceramic/polymer nanocomposite dielectric capacitor with enhanced energy storage performance. Polym. Eng. Sci..

[89] Li H., Fu Y., Alhashmialameer D., Thabet H.K., Zhang P., Wang C., Zhu K., Huang M., Guo Z., Dang F. (2022). Lattice distortion embedded core–shell nanoparticle through epitaxial growth barium titanate shell on the strontium titanate core with enhanced dielectric response. Adv. Compos. Hybrid Mater..

[90] Yang K., Huang X., Zhu M., Xie L., Tanaka T., Jiang P. (2014). Combining RAFT Polymerization and Thiol–Ene Click Reaction for Core–Shell Structured Polymer@BaTiO_3_ Nanodielectrics with High Dielectric Constant, Low Dielectric Loss, and High Energy Storage Capability. ACS Appl. Mater. Interfaces.

[91] Wu W., Ren T., Liu X., Huai K., Cui X., Wei H., Hu J., Xia Y., Huang S., Fu K. (2022). Electric field-assisted preparation of PANI/TPU all-organic composites with enhanced dielectric permittivity and anisotropic optical properties. Polym. Eng. Sci..

[92] Wei H., Yuan Y., Ren T., Zhou L., Liu X., Saeed H.A.M., Jin P., Chen Y. (2022). High-Dielectric PVP@PANI/PDMS Composites Fabricated via an Electric Field-Assisted Approach. Polymers.

[93] Wang Q., Liu X., Qiang Z., Hu Z., Cui X., Wei H., Hu J., Xia Y., Huang S., Zhang J. (2022). Cellulose nanocrystal enhanced, high dielectric 3D printing composite resin for energy applications. Compos. Sci. Technol..

[94] Dash K., Hota N.K., Sahoo B.P. (2020). Fabrication of thermoplastic polyurethane and polyaniline conductive blend with improved mechanical, thermal and excellent dielectric properties: Exploring the effect of ultralow-level loading of SWCNT and temperature. J. Mater. Sci..

[95] Zhang Q., Jiang Y., Yu E., Yang H. (2018). Significantly enhanced dielectric properties of P(VDF-HFP) composite films filled with core-shell BaTiO_3_@ PANI nanoparticles. Surf. Coat. Technol..

[96] Rahnamol A.M., Gopalakrishnan J. (2020). Improved dielectric and dynamic mechanical properties of epoxy/polyaniline nanorod/ *in situ* reduced graphene oxide hybrid nanocomposites. Polym. Compos..

[97] Khouaja A., Koubaa A., Ben Daly H. (2021). Dielectric properties and thermal stability of cellulose high-density polyethylene bio-based composites. Ind. Crop. Prod..

[98] Guo Q., Xue Q., Wu T., Pan X., Zhang J., Li X., Zhu L. (2016). Excellent dielectric properties of PVDF-based composites filled with carbonized PAN/PEG copolymer fibers. Compos. Part A Appl. Sci. Manuf..

[99] Wang P., Yin Y., Fang L., He J., Wang Y., Cai H., Yang Q., Shi Z., Xiong C. (2023). Flexible cellulose/PVDF composite films with improved breakdown strength and energy density for dielectric capacitors. Compos. Part A Appl. Sci. Manuf..

[100] Chen Y., Liu Y., Yang J., Zhang B., Hu Z., Wang Q., Wu W., Shang Y., Xia Y., Duan Y. (2019). Fabrication of high dielectric permittivity polymer composites by architecting aligned micro-enhanced-zones of ultralow content graphene using electric fields. Mater. Today Commun..

[101] Chen Y., Liu Y., Xia Y., Liu X., Qiang Z., Yang J., Zhang B., Hu Z., Wang Q., Wu W. (2020). Electric Field-Induced Assembly and Alignment of Silver-Coated Cellulose for Polymer Composite Films with Enhanced Dielectric Permittivity and Anisotropic Light Transmission. ACS Appl. Mater. Interfaces.

[102] Tang H., Lin Y., Sodano H.A. (2012). Enhanced Energy Storage in Nanocomposite Capacitors through Aligned PZT Nanowires by Uniaxial Strain Assembly. Adv. Energy Mater..

[103] Zhang X., Jiang J., Shen Z., Dan Z., Li M., Lin Y., Nan C., Chen L., Shen Y. (2018). Polymer Nanocomposites with Ultrahigh Energy Density and High Discharge Efficiency by Modulating their Nanostructures in Three Dimensions. Adv. Mater..

[104] Zhang Y., Zhang C., Feng Y., Zhang T., Chen Q., Chi Q., Liu L., Li G., Cui Y., Wang X. (2019). Excellent energy storage performance and thermal property of polymer-based composite induced by multifunctional one-dimensional nanofibers oriented in-plane direction. Nano Energy.

[105] Luo H., Zhou X., Ellingford C., Zhang Y., Chen S., Zhou K., Zhang D., Bowen C.R., Wan C. (2019). Interface design for high energy density polymer nanocomposites. Chem. Soc. Rev..

[106] Huang Y., Huang X., Schadler L.S., He J., Jiang P. (2016). Core@ Double-Shell Structured Nanocomposites: A Route to High Dielectric Constant and Low Loss Material. ACS Appl. Mater. Interfaces.

[107] Wang D., Bao Y., Zha J.-W., Zhao J., Dang Z.-M., Hu G.-H. (2012). Improved Dielectric Properties of Nanocomposites Based on Poly(vinylidene fluoride) and Poly(vinyl alcohol)-Functionalized Graphene. ACS Appl. Mater. Interfaces.

[108] Yang K., Huang X., Xie L., Wu C., Jiang P., Tanaka T. (2012). Core-Shell Structured Polystyrene/BaTiO_3_ Hybrid Nanodielectrics Prepared by In Situ RAFT Polymerization: A Route to High Dielectric Constant and Low Loss Materials with Weak Frequency Dependence. Macromol. Rapid Commun..

[109] Wu W., Liu X., Qiang Z., Yang J., Liu Y., Huai K., Zhang B., Jin S., Xia Y., Fu K.K. (2021). Inserting insulating barriers into conductive particle channels: A new paradigm for fabricating polymer composites with high dielectric permittivity and low dielectric loss. Compos. Sci. Technol..

[110] Abutalib M.M., Rajeh A. (2021). Boosting optical and electrical characteristics of polyvinyl alcohol/carboxymethyl cellulose nanocomposites by GNPs/MWCNTs fillers as an application in energy storage devices. Int. J. Energy Res..

